# Supplementary material for: A metagenomic analysis of the phase 2 Anopheles gambiae 1000 genomes dataset reveals a wide diversity of cobionts associated with field collected mosquitoes
Source: Commun Biol. 2024 May 30;7:667. doi: 10.1038/s42003-024-06337-9 (PMC11139907; doi:10.1038/s42003-024-06337-9)
Supplement: Supplementary file 2 — Supplementary Information [file 42003_2024_6337_MOESM2_ESM.pdf]

A metagenomic analysis of the phase 2 *Anopheles gambiae* 1000 genomes dataset reveals a wide diversity of cobionts associated with field collected mosquitoes

Andrzej Pastusiak[1], Michael R. Reddy[1*], Xiaoji Chen[1], Isaiah Hoyer[1], Jack Dorman[2], Mary E. Gebhardt[3], Giovanna Carpi[2], Douglas E. Norris[3], James M. Pipas[4] and Ethan K. Jackson[1]

[1]Microsoft Premonition, Microsoft Research, Redmond, WA, 98052 USA

[2]Department of Biological Sciences, Purdue University, West Lafayette, IN 47907 USA

[3]The W. Harry Feinstone Department of Molecular Microbiology and Immunology, Johns Hopkins Malaria Research Institute, Johns Hopkins Bloomberg School of Public Health, Baltimore, MD, 21205 USA

[4]Department of Biological Sciences, University of Pittsburgh, Pittsburgh, PA 15260 USA

*Corresponding Author:

Email: michael.reddy@microsoft.com (MRR)

SUPPLEMENTARY INFORMATION

Supplementary Note 1- Detection of viral and bacteriophage species by the BMM and Integrator pipelines.

Application of the BMM pipeline to the Ag1000G sequence dataset revealed 2,039,560 reads (0.001%) aligning to bacterial and eukaryotic viral species (Supplementary Data 4- “All viral species detected”). The pipeline called 80 species of eukaryotic viruses and bacteriophage distributed across 223 of the 1,142 specimens. Integrator was used to examine the open reading frame (ORF) structure and amino acid sequence identity of contigs assembled from all 1,142 specimens. This was followed by Genetic Signature Analysis, a systematized manual inspection procedure designed to identify contaminants and computational mis-assignments and to confirm viral presence. These steps result in 33 specimens containing probable novel bacteriophage, and sixteen specimens containing eukaryotic viruses. Thus, viral sequences were detected in a small minority of mosquitoes. Following removal of contaminants and misassignments, three eukaryotic viral species were determined to be present in sixteen specimens. Hepatitis B virus (HBV) was present in fifteen specimens, primate erythroparvovirus-1 in three specimens, and ungulate erythroparvovirus-1 in a single specimen. The mosquitoes containing HBV and primate erythroparvovirus-1 also contained human blood, while the mosquito in which ungulate erythroparvovirus-1 was identified contained bovine blood. Nearly all the authenticated bacteriophage identifications seem novel, distantly related to the known phage in sequence databases.

Supplementary Note 2- Presence of HBV.

BMM detected HBV sequences in fifteen specimens (Supplementary Data 4- “All viral

species detected”; Fig. S3). Sequence reads from these specimens were assembled and probed with Integrator. Integrator reported contigs with amino acid alignments consistent with HBV in twelve of these specimens. Integrator did not detect HBV-related sequences in any of the other 1,127 mosquitoes. Some of the contigs represent full-length assemblies of the HBV genome, while others have varying amounts of coverage (Fig. S3). The order and protein identify of each ORF was subjected to BLASTp. In each case, the ORF-identity and the gene order were consistent with HBV. Alignment of the HBV nucleotide sequences present in the fifteen specimens revealed that each contained unique polymorphisms with respect to the HBV reference strain. Finally, we examined the bloodmeal and metadata associated with each of the specimens harboring HBV. All fifteen HBV-positive mosquitoes contained human blood, but the number of human sequence reads varied greatly across the specimens. This could be partly due to variations in the time between feeding and collection and/or preservation. A further complication is our discovery that all the mosquito specimens contained at least some human sequences, suggesting post-feeding contamination.

Supplementary Note 3. Presence of ungulate erythroparvovirus-1.

BMM detected ungulate erythroparvovirus-1 sequences in a single sample (Supplementary Data 4- “All viral species detected” and “ERS248730- BMM vs Kraken2” and Fig. S6 a,b). Assembly of sequences obtained from this mosquito yielded a single contig consistent with the genome structure of a parvovirus. These viral ORFs had high amino acid identity with ungulate erythroparvovirus-1 (Fig. S4). The mosquito harboring these viral sequences also contained many bovine sequence reads, consistent with a cow bloodmeal. This analysis coupled with the fact that ungulate

erythroparvovirus-1 is tropic for erythrocytes, and thus would be expected to be present in blood, provide strong evidence for capture of this virus from a viremic host.

Supplementary Note 4- Presence of primate erythroparvovirus-1.

BMM detected primate erythroparvovirus-1 sequences in three specimens (Supplementary Data 4- “All viral species detected”). While sequence coverage was spread across the viral genome (Fig. S5) no viral contigs were obtained from these specimens. All three mosquito specimens contained reads assigned to human DNA.

Supplementary Note 5- Comparison of Premonition BMM and Kraken2 pipelines for resolving a complex specimen.

We compared the BMM pipeline against another widely used metagenomics pipeline, the Kraken2 metagenomic analysis suite.[1] To do this, both pipelines were tasked with resolving the composition of one of the most “complex” specimens in the dataset, specimen ERS248730. This specimen was initially identified by the BMM pipeline to contain material derived from a mosquito, a vertebrate blood meal and microbial content including bacteria and viruses (Fig. S6; Supplementary Data 4- “ERS248730- BMM vs Kraken2”). The results generated by each pipeline were highly congruent with respect to the numbers of reads and percentages of total reads assigned. The BMM pipeline assigned 72.0% of all reads to the *Anopheles gambiae* species complex mosquitoes with the majority assigned to *Anopheles gambiae* (46%). Vertebrate reads constituted 24.5% of total assigned reads with 98% of those reads assigned to a single ungulate host, *Bos taurus* (Fig. S6a). The remainder of reads were distributed among viral, bacterial, and other, not clean, and unassigned taxa representing 0.02%, 0.002%, 0.6%, 1.6%, and 1.3% respectively of total reads (Supplementary Data 4- “ERS248730- BMM

vs Kraken2.”). All viral reads were assigned to ungulate erythroparvovirus-1. and represent 100% coverage of the reference genome (Fig. S4). Coverage is defined as the total fraction of reference genome locations for which at least one read is assigned to that location. The Kraken2 pipeline assigned 72.8% of all reads to the *Anopheles gambiae* species complex with 48% assigned to *Anopheles gambiae*. 24.7% of all reads were assigned to bovid hosts with 16% and 5% assigned to *Bos taurus* and *Bos indicus* respectively. Reads assigned to ungulate erythroparvovirus-1 constituted 0.008% of the total number of reads (Fig. S6b). The remainder of reads were represented by other taxa (1.3%) or were unassigned (1.2%) by the Kraken2 pipeline (Supplementary Data 4- “ERS248730- BMM vs Kraken2.”).

The largest challenge with performing the analysis using the Kraken2 pipeline was the preparation of a reference database. The pre-built Kraken2 databases available online did not contain mosquito and mammalian reference sequences found in the RefSeq and Genbank databases used by the BMM pipeline. It took approximately ten days to build the database and required a virtual machine with eleven terabytes of memory. Such a computation would not have been possible in 2018 when we performed our initial analysis of the Phase I Ag1000G dataset with the Premonition BMM pipeline. The current databases have grown six-fold since the original analysis was performed. The largest machines available at that time had only about four terabytes of memory. The resulting Kraken database file size is about one terabyte. After building the Kraken2 reference database based on the same FASTA sequences used with the Premonition BMM pipeline, 99% of the reads were found to align.

Supplementary Note 6- Novel bacteriophage.

BMM identified sequences aligning to known bacteriophage in 177 specimens (Supplementary Data 4- “All viral species detected” and “Potential novel bacteriophage”). In most cases the sequence coverage of each bacteriophage species was low suggesting that most represent novel agents with low similarity to known viruses. This conclusion is supported by analysis with Integrator which revealed numerous contigs containing ORFs distantly related to known bacteriophage. Multiple contigs with identities assigned to different known bacteriophage species were often present in the same mosquito specimen. Further analysis is required to determine if these cases represent a single novel bacteriophage or if multiple novel bacteriophages are present in the same specimen. Because bacteriophage require bacterial hosts, we determined whether bacteria sequences were present in those specimens containing bacteriophage.

Supplementary Note 7- Viruses with RNA genomes.

The BMM identified five eukaryotic viruses that have RNA genomes: Avian leukosis virus, Enterovirus A, Influenza A, Influenza B, and HIV. All of these were determined to be contaminants, most likely originating from sequencing reagents or sequencer flow cells. PickAxe is a virus detection pipeline that uses both nucleic acid identity and protein amino acid sequence identity to uncover known and novel viruses from metagenomic NGS data.[2] PickAxe detected ORFs related to two additional viral species, Karumba virus, a Flavivirus, and *An. annulipes* orbivirus, a Reovirus.[3,4] In each case amino acid alignment by BLASTp indicated that the viral sequences likely represented novel viruses that are distant relatives of these species. In addition,

sequences related but not identical to three unclassified RNA viruses were detected by PickAxe. These included Wilkie parti-like virus 2, Wilkie qin-like virus, and Wuhan insect virus 23. Analysis of the ORF structure of each contig representing these five viruses indicated that none were consistent with a viral genome. This observation, coupled with the fact that RNA should not be detectable by DNA sequencing, lead us to speculate that these contigs identify partial integrated viral genomes.

SUPPLEMENTARY FIGURES

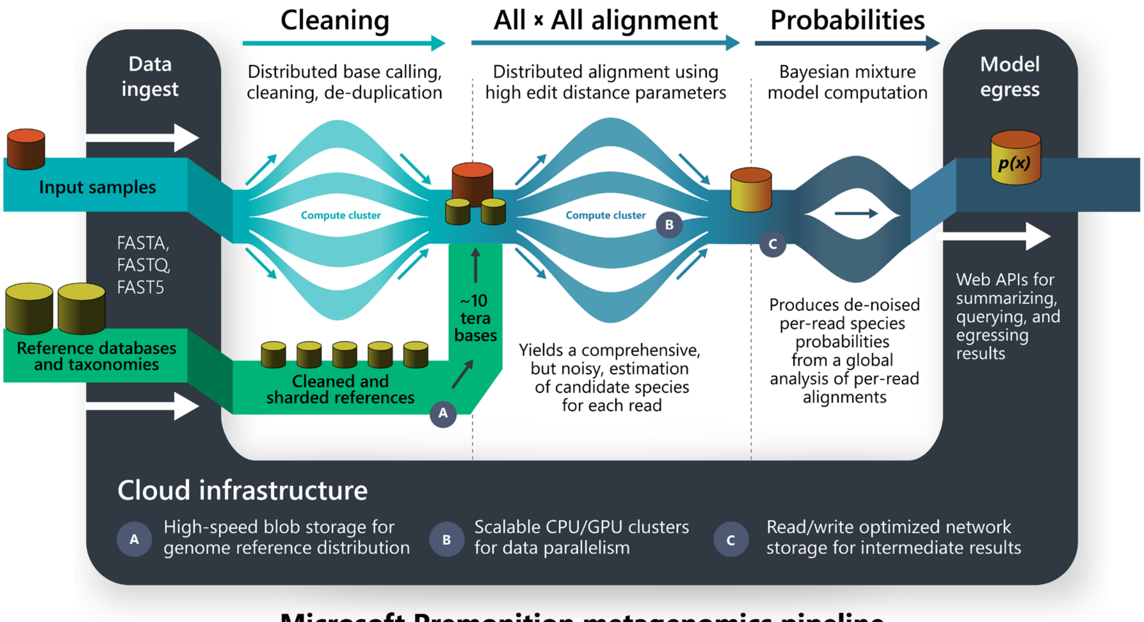

Microsoft Premonition metagenomics pipeline

Fig. S1. Microsoft Premonition metagenomics pipeline. Summarizes the data flow and cloud architecture of the metagenomics pipeline. Of note is the All x All alignment step that requires dynamically scalable cloud compute to efficiently achieve. Bayesian mixture model computation uses a global expectation-maximization algorithm and fine-tuned heuristics to convert candidate alignments into a probability distribution of source species for each read. Output models are large, so web APIs provide capabilities to query and summarize models to avoid downloading the entire BMM.

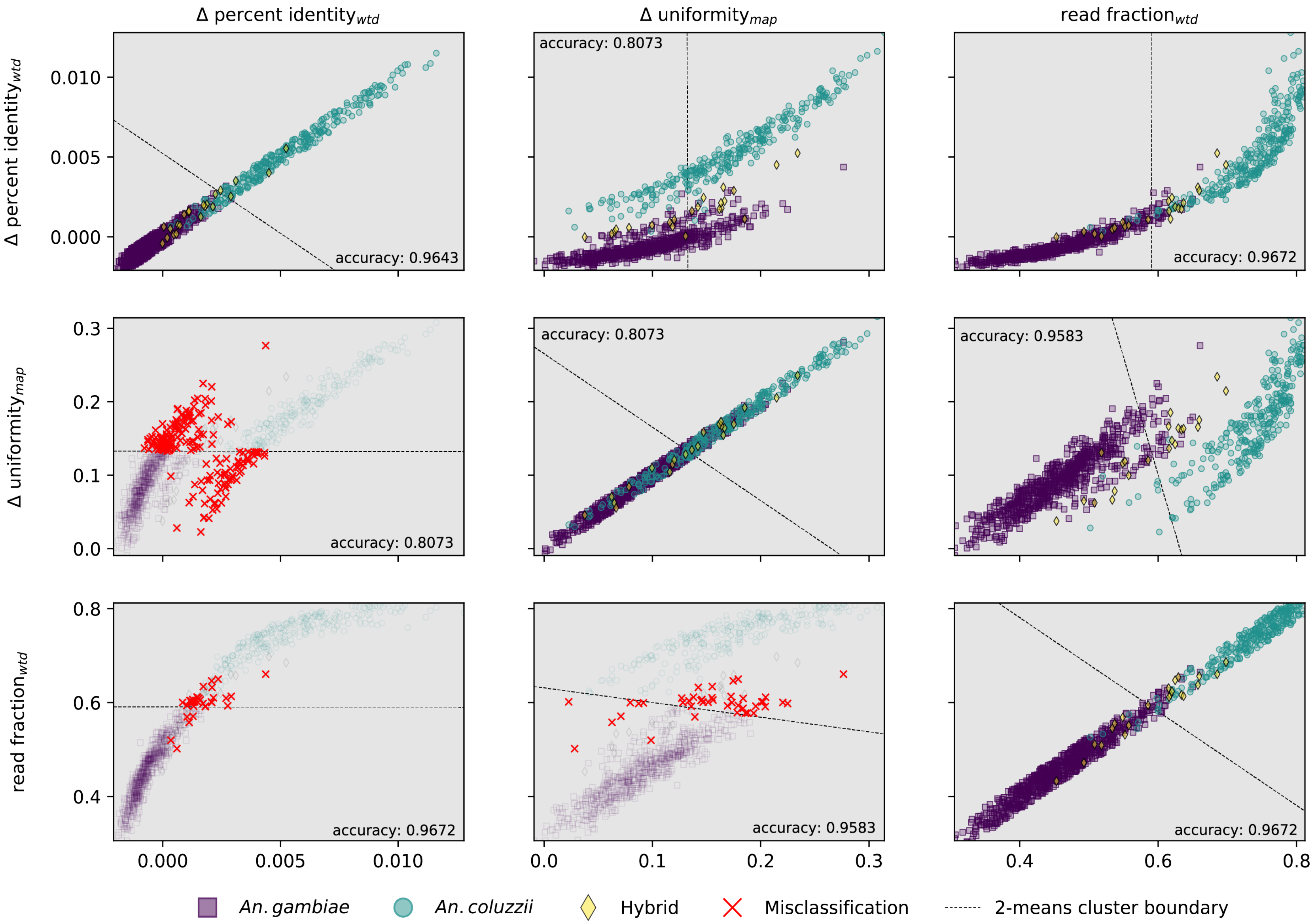

Δ percent identity$_{wtd}$
Δ uniformity$_{map}$
read fraction$_{wtd}$
accuracy: 0.9643
accuracy: 0.8073
accuracy: 0.9672
accuracy: 0.8073
accuracy: 0.8073
accuracy: 0.9583
accuracy: 0.9672
accuracy: 0.9583
accuracy: 0.9672
0.010
0.005
0.000
0.3
0.2
0.1
0.0
0.8
0.6
0.4
0.000
0.005
0.010
0.0
0.1
0.2
0.3
0.4
0.6
0.8
An. gambiae
An. coluzzii
Hybrid
Misclassification
2-means cluster boundary

Fig. S2. Straightforward clustering produces accurate species calls. This figure shows that simple two-means clustering of various BMM statistics accurately separates confirmed *An. gambiae* and *An. coluzzi* specimens. The features shown are: 1) Δpercent identity$_{wtd}$, which is the difference between the average percent identity to *An. gambiae* reference GCA_000150785.1 and the average percent identity to *An. coluzzi* reference GCA_000150765.1, *except* that read-level %id scores are weighted by read probabilities i.e. less probable reads reduce overall %id; 2) read fraction$_{wtd}$, which is the sum of read-level probabilities assigned to *An. gambiae* divided by the sum of read-level probabilities assigned *An. coluzzi*, essentially a weighted ratio of reads assigned to each genome, and 3) Δuniformity$_{map}$, which estimates the difference in uniformity of genome coverage, after first computing genome coverage by assuming reads comes genomes of highest probability i.e. applying a maximum a posteriori (*map*) decision rule to reads. Features that increase with %id and total number of assigned reads all perform well. Coverage related features i.e. Δuniformity$_{map}$ show decreased efficacy, presumably because both genomes are well-covered and SNPs are spread throughout the genomes. The hybrid specimens are not counted in accuracy statistics, but their BMM features are shown.

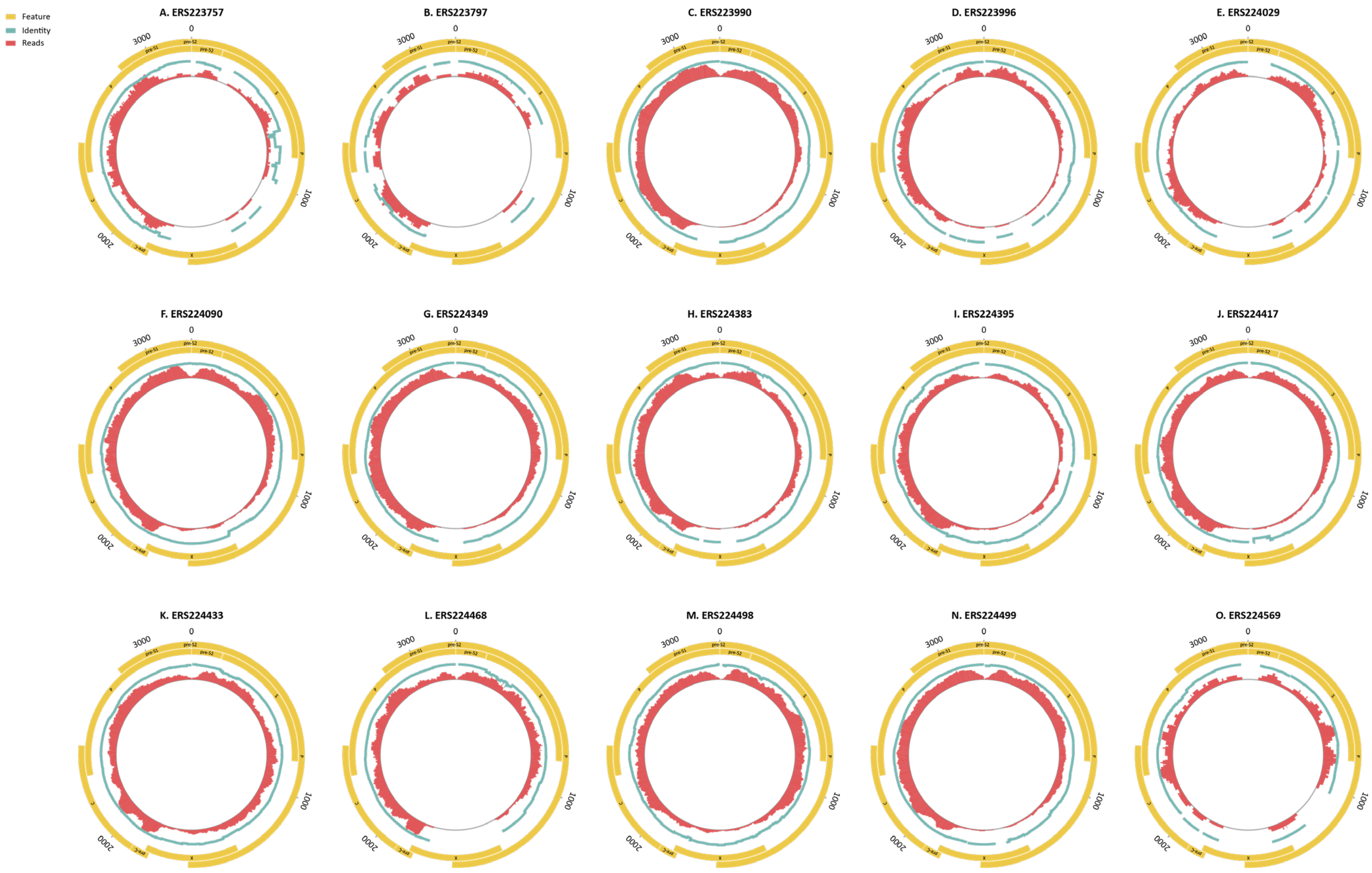

Fig. S3. HBV Coverage Maps. Panels **A – O** show the sequence coverage maps of HBV found in each of 15 specimens. Yellow illustrates the HBV open reading frames encoding viral proteins. Redish-orange and blue indicate the sequence coverage depth and percent identity respectively.

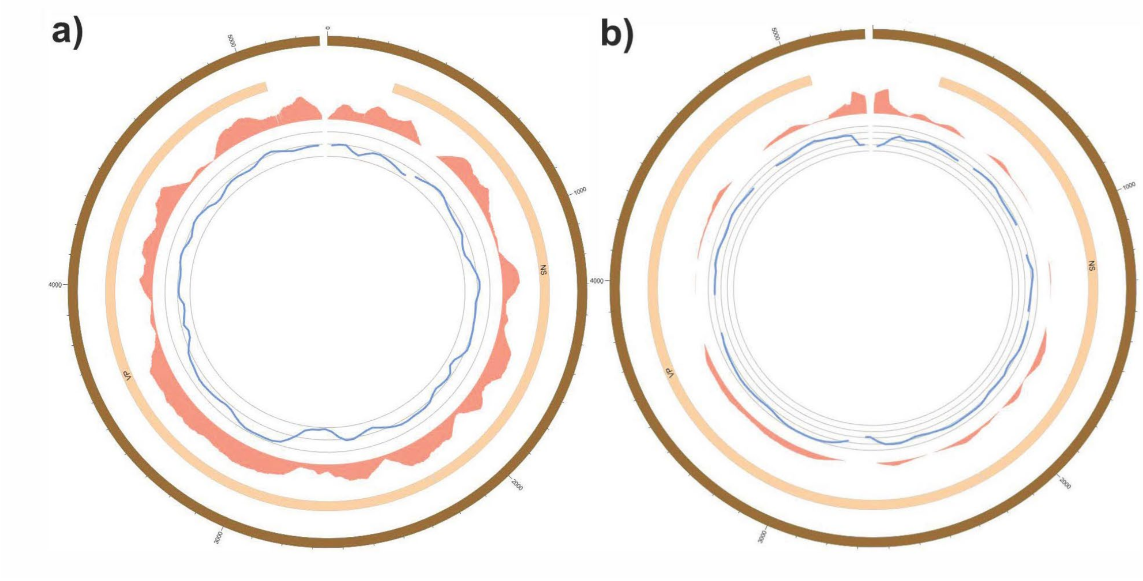

Fig. S4. Coverage maps of ungulate erythroparvovirus-1 identified in specimen ERS248730. a) Premonition BMM pipeline output. **b)** Kraken2 pipeline output. Redish-orange represents depth of coverage and blue represents the average percent identity of all reads assigned to a reference genome. Brown represents the ungulate erythroparvovirus-1 DNA genome. Light brown illustrates the ungulate erythroparvovirus-1 open reading frames encoding viral proteins.

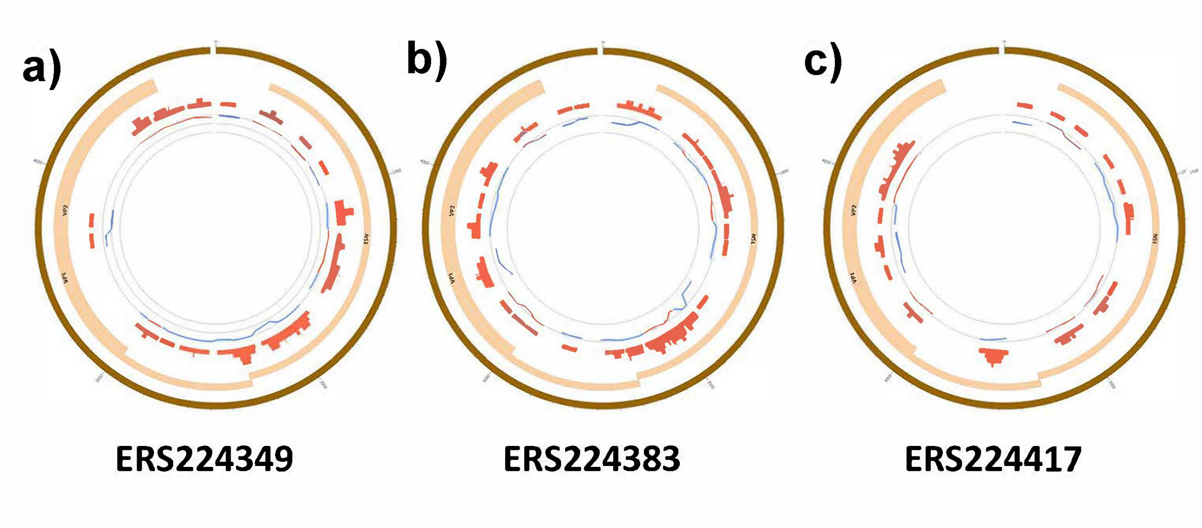

Fig. S5. Coverage maps of primate erythroparvovirus-1. Panels **a-c** show the sequence coverage maps of primate erythroparvovirus-1 found in each of 15 specimens. Brown represents the primate erythroparvovirus-1 genome DNA genome. Light brown illustrates the primate erythroparvovirus-1 open reading frames encoding viral proteins. Redish-orange and blue indicate the sequence coverage and depth.

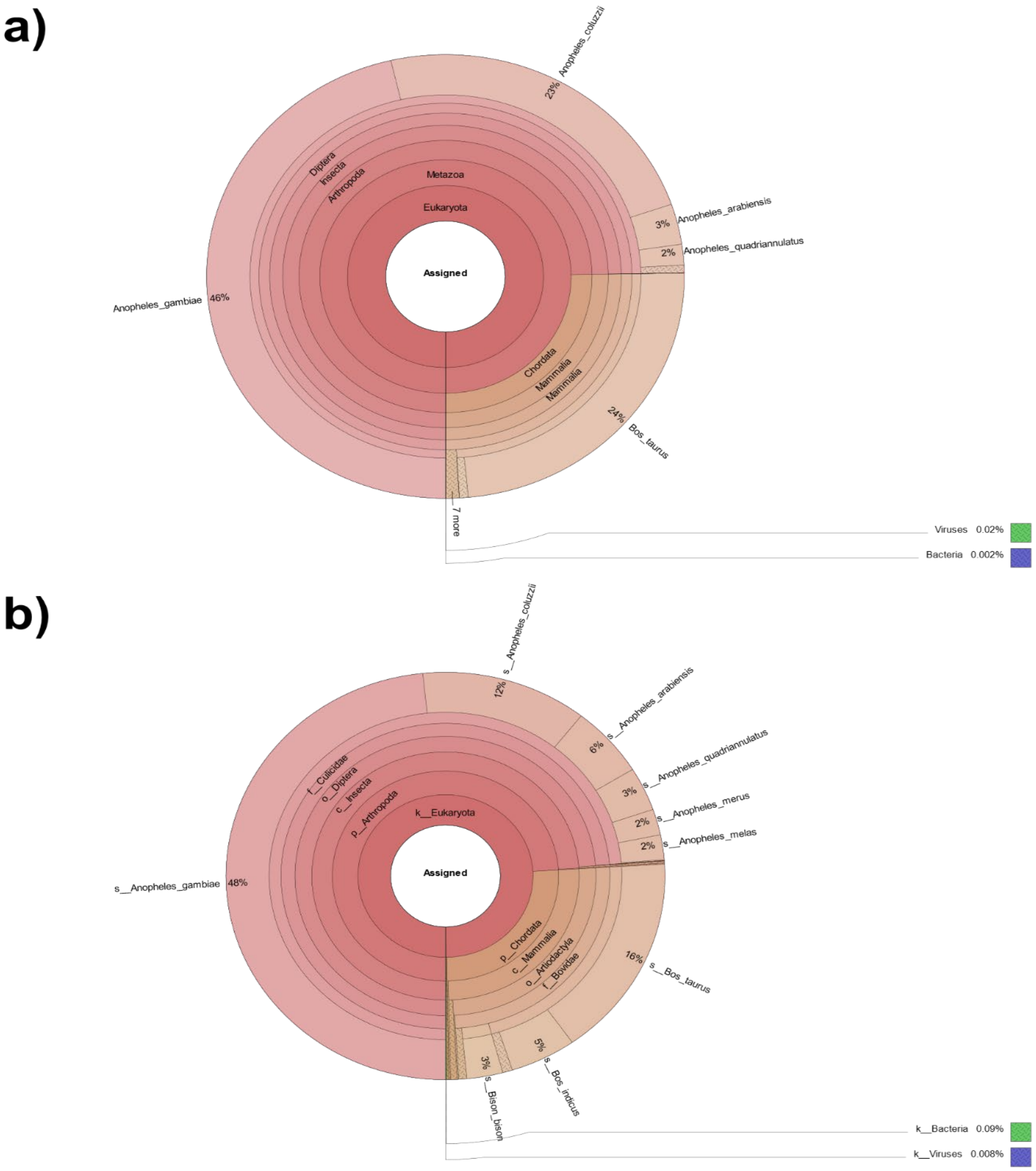

Fig. S6. BMM and Kraken2 pipeline output for specimen ERS248730 a) Krona plot of BMM pipeline results indicating the percentage of total reads assigned to *Anopheles gambiae* species complex mosquitoes, Bovid, viral and bacterial taxa identified within specimen ERS248730. **b)** Krona plot of Kraken2 pipeline results indicating percentage of total reads assigned to *Anopheles gambiae* species complex mosquito, Bovid, bacterial and viral taxa identified within specimen ERS248730.

SUPPLEMENTARY INFORMATION REFERENCES:

1. Lu, J. *et al.* Metagenome analysis using the Kraken software suite. *Nature Protocols* **17**, 2815–2839 (2022).
2. Cantalupo, P. G., Katz, J. P. & Pipas, J. M. Viral sequences in human cancer. *Virology* **513**, 208–216 (2018).
3. Cantalupo, P. G. *et al.* Raw sewage harbors diverse viral populations. *MBio,* **2**, 10.1128/mbio.00180-11 (2011).
4. Cantalupo, P. G. & Pipas, J. M. Detecting viral sequences in NGS data. *Current Opinion in Virology* **39**, 41–48 (2019).
