# Supplementary material for: A metagenomic analysis of the phase 2 Anopheles gambiae 1000 genomes dataset reveals a wide diversity of cobionts associated with field collected mosquitoes
Source: Commun Biol. 2024 May 30;7:667. doi: 10.1038/s42003-024-06337-9 (PMC11139907; doi:10.1038/s42003-024-06337-9)
Supplement: Supplementary file 3 — Description of Additional Supplementary Files [file 42003_2024_6337_MOESM3_ESM.pdf]

Description of Additional Supplementary Files

File name: Supplementary Data 1

Description: Source data for Figures 1-4 & 6

File name: Supplementary Data 2

Description: Source Data for Figure 5a

File name: Supplementary Data 3

Description: Source Data for Figure 5b

File name: Supplementary Data 4

Description: Supplementary Tables S1-S8 combined in single .xlsx file
