# Supplementary material for: A metagenomic analysis of the phase 2 Anopheles gambiae 1000 genomes dataset reveals a wide diversity of cobionts associated with field collected mosquitoes
Source: Commun Biol. 2024 May 30;7:667. doi: 10.1038/s42003-024-06337-9 (PMC11139907; doi:10.1038/s42003-024-06337-9)
Supplement: Supplementary file 8 — Reporting Summary [file 42003_2024_6337_MOESM8_ESM.pdf]

nature portfolio

Corresponding author(s): Michael R. Reddy
Last updated by author(s): 2024-05-07

Reporting Summary

Nature Portfolio wishes to improve the reproducibility of the work that we publish. This form provides structure for consistency and transparency in reporting. For further information on Nature Portfolio policies, see our Editorial Policies and the Editorial Policy Checklist.

Statistics

For all statistical analyses, confirm that the following items are present in the figure legend, table legend, main text, or Methods section.

n/a	Confirmed	
☐	☒	The exact sample size (*n*) for each experimental group/condition, given as a discrete number and unit of measurement
☐	☒	A statement on whether measurements were taken from distinct samples or whether the same sample was measured repeatedly
☐	☒	The statistical test(s) used AND whether they are one- or two-sided<br>*Only common tests should be described solely by name; describe more complex techniques in the Methods section.*
☐	☒	A description of all covariates tested
☐	☒	A description of any assumptions or corrections, such as tests of normality and adjustment for multiple comparisons
☐	☒	A full description of the statistical parameters including central tendency (e.g. means) or other basic estimates (e.g. regression coefficient) AND variation (e.g. standard deviation) or associated estimates of uncertainty (e.g. confidence intervals)
☐	☒	For null hypothesis testing, the test statistic (e.g. *F*, *t*, *r*) with confidence intervals, effect sizes, degrees of freedom and *P* value noted<br>*Give P values as exact values whenever suitable.*
☐	☒	For Bayesian analysis, information on the choice of priors and Markov chain Monte Carlo settings
☐	☒	For hierarchical and complex designs, identification of the appropriate level for tests and full reporting of outcomes
☐	☒	Estimates of effect sizes (e.g. Cohen's *d*, Pearson's *r*), indicating how they were calculated

Our web collection on statistics for biologists contains articles on many of the points above.

Software and code

Policy information about availability of computer code

Data collection	Access to read-level data is managed by the Ag1000 Genome Consortium (terms and conditions apply) and should be requested directly from the Ag1000 Genome Consortium.
Data analysis	The Microsoft Premonition pipeline is a proprietary cloud service, and APIs to access this service are made available to select partners through the Microsoft Premonition Early Access Program (terms and conditions apply). If needed to assist reviewers, authors will provide the computed mixture model statistics, aggregated at the sample level as a data artifact upon request.

For manuscripts utilizing custom algorithms or software that are central to the research but not yet described in published literature, software must be made available to editors and reviewers. We strongly encourage code deposition in a community repository (e.g. GitHub). See the Nature Portfolio guidelines for submitting code & software for further information.

Data

Policy information about availability of data

All manuscripts must include a data availability statement. This statement should provide the following information, where applicable:

- Accession codes, unique identifiers, or web links for publicly available datasets
- A description of any restrictions on data availability
- For clinical datasets or third party data, please ensure that the statement adheres to our policy

DATA AVAILABILITY STATEMENT

March 2021

The datasets analyzed in the current study are available via the Anopheles gambiae 1000 Genomes Consortium website:
• Ag1000G phase 1 AR3.1 data release: https://www.malariagen.net/data/ag1000g-phase1-ar3.1
• Ag1000G phase 2 AR1 data release: http://www.malariagen.net/data/ag1000g-phase2-ar1
All data generated during this study are included in this published article (and its Supplementary Information files).

Human research participants

Policy information about studies involving human research participants and Sex and Gender in Research.

Reporting on sex and gender	N/A
Population characteristics	N/A
Recruitment	N/A
Ethics oversight	N/A

Note that full information on the approval of the study protocol must also be provided in the manuscript.

Field-specific reporting

Please select the one below that is the best fit for your research. If you are not sure, read the appropriate sections before making your selection.

☒ Life sciences ☐ Behavioural & social sciences ☐ Ecological, evolutionary & environmental sciences

For a reference copy of the document with all sections, see nature.com/documents/nr-reporting-summary-flat.pdf

Life sciences study design

All studies must disclose on these points even when the disclosure is negative.

Sample size	Pre-defined dataset analyzed. Whole genome sequences (n=1,142 An. gambiae mosquito genomes) are available via the Anopheles gambiae 1000 Genomes Consortium website.
Data exclusions	No data was excluded
Replication	All attempts at replication were successful
Randomization	Randomization does not apply to this analysis
Blinding	Blinding was not conducted. Full dataset including metadata are available via the Anopheles gambiae 1000 Genomes Consortium website

Reporting for specific materials, systems and methods

We require information from authors about some types of materials, experimental systems and methods used in many studies. Here, indicate whether each material, system or method listed is relevant to your study. If you are not sure if a list item applies to your research, read the appropriate section before selecting a response.

Materials & experimental systems

n/a	Involved in the study	
☒	☐	Antibodies
☒	☐	Eukaryotic cell lines
☒	☐	Palaeontology and archaeology
☒	☐	Animals and other organisms
☒	☐	Clinical data
☒	☐	Dual use research of concern

Methods

n/a	Involved in the study	
☒	☐	ChIP-seq
☒	☐	Flow cytometry
☒	☐	MRI-based neuroimaging
